# Virulence and phylogenetic groups of *Escherichia coli* cultured from raw sewage in Kuwait

**DOI:** 10.1186/s13099-022-00490-4

**Published:** 2022-04-26

**Authors:** Mahdi A. Redha, Noura Al Sweih, M. John Albert

**Affiliations:** Department of Microbiology, Faculty of Medicine, Kuwait University, Jabriya, Kuwait

**Keywords:** Sewage, *Escherichia coli*, Phylogenetic grouping, Diarrheagenic *E. coli*, Extra-intestinal pathogenic *E. coli*

## Abstract

In Kuwait, some untreated sewage is discharged into the sea which poses health risks. Therefore, we determined the virulence traits and the phylogenetic groups of *E. coli* cultured from raw sewage. Sewage was collected once every month for 12 months with culturing of a total of 140 *E. coli* isolates. *E. coli* was typed by the methods of Clermont. The five pathotypes of diarrheagenic *E. coli* (DEC), and extra-intestinal pathogenic *E. coli* (ExPEC) were detected by specific PCR assays. Four virulence genes which correlate with pathogenicity in animal models, were used for the first time for detection of ExPEC—*vat* (vacuolating auto-transporter toxin), *fyuA* (yersiniabactin receptor)*, chuA* (heme-binding protein)*,* and *yfcV* (major subunit of putative chaperon-usher fimbria). Most *E. coli* belonged to phylogenetic groups A (65[45%]) and B1 (34[24.3%]). Three (2.1%) isolates were DEC, while 14 (10%) isolates were ExPEC mostly in group B2 (57.1%). A relatively high prevalence of ExPEC in sewage has public health implications.

**Supplementary Information:**

The online version contains supplementary material available at 10.1186/s13099-022-00490-4.

Sewage is the wastewater consisting of human excreta, wash water, and industrial and agricultural wastes. It is rich in microbes, including bacteria, fungi, parasites, and viruses. The origin of microbes is intestinal (from humans, animals, birds, etc.) and soil. In Kuwait, about 75% of sewage is treated and the remainder is discharged into the sea untreated [1]. Coastal waters are commonly used for recreational purposes in Kuwait.

The discharge of untreated sewage into the coastal waters has health implications for the people who use these waters for recreation. *E. coli* forms the predominant facultative anaerobic flora of the feces, and hence, it is used as an indicator organism for fecal pollution. Drug-resistant fecal *E. coli* has been previously cultured from the coastal waters of Kuwait [2]. *E. coli* can cause both intestinal and extra-intestinal infections [3]. Therefore, we studied *E. coli* isolates cultured from raw sewage in Kuwait for their virulence properties associated with diarrheal disease and extra-intestinal infections. We also did phylogenetic grouping of the isolates. We defined extra-intestinal pathogenic *E. coli* (ExPEC) by a set of genes that has not been previously used to screen *E*. *coli* from sewage. In addition, we sought all the five pathotypes of diarrheagenic *E. coli* (DEC) – enteropathogenic *E. coli* (EPEC), enterotoxigenic *E. coli* (ETEC), enteroinvasive *E. coli* (EIEC), shiga toxin-producing *E. coli* (STEC) and enteroaggregative *E. coli* (EAEC) [3]—which to our knowledge, has rarely been done, in previous studies of *E. coli* from sewage.

The Underworlds Project is a collaborative project among MIT-Harvard in the USA, Kuwait Institute for Scientific Research (KISR), Kuwait University, Kuwait Ministry of Public Works, Kuwait Ministry of Health, Kuwait Ministry of Defense, and Kuwait Department of Water and Electricity. This pilot project aims to set up for the State of Kuwait an eventual monitoring system for medicines, phthalates, bioterrorism agents, explosives, antimicrobial resistance genes, bacterial markers of obesity, and infectious disease epidemics (for e.g. due to *Salmonella* spp., norovirus, rotavirus, influenza virus etc.) by regular analysis of sewage at certain locations. We utilized the *E. coli* isolates cultured from this project for the present study.

A sewage sample (1000 ml) was collected once every month for 12 months (May 2018 to April 2019) from three sites – Jabriya (latitude: 29.318057, longitude: 48.025805), Zahraa (latitude: 47.99263, longitude**:** 29.26725) and Hateen (latitude: 29.2842, longitude 48.0184) – with a total of 36 samples. Jabriya is about 7.8 km southeast of Kuwait City, and Zahraa and Hateen are about 11.5 km and 10.8 km respectively south of Kuwait City. Distance between Jabriya and Zahraa is 5.9 km; that between Jabriya and Hateen is 4.1 km; and that between Zahraa and Hateen is 2.1 km. The three sites of sample collection are indicated in Fig. 1. These locations were selected for ease of sample collection. The numbers of people living in these locations at the end of 2018 were: 78,026 (Jabriya), 32,064 (Zahraa) and 21,557 (Hateen) (https://www.citypopulation.de/en/kuwait/admin/). Immediately after the sample was collected, it was transported to KISR in an insulated Styrofoam box with icepacks, for initial processing. After allowing the large particles to settle, a 10.0 ml sample was filtered through a 0.2 μm pore-size membrane (Sartorius). The membrane was immediately transported in cold Styrofoam box to the Enteric Microbiology Laboratory, Faculty of Medicine, Kuwait University, Jabriya, Kuwait, where further processing was done. The membrane was cut into small pieces and vortexed in 1.0 ml of phosphate buffered saline (PBS, pH 7.2). This PBS washing was cultured on m-FC agar (Oxoid) which was incubated at 42 °C for 24 h [4]. Different morphotypes of blue colonies (one to ten) of presumed *E. coli* were picked and screened for substrate utilization in the API-20E biochemical strip (bioMerieux). Colonies identified as *E. coli* were stocked in tryptic soy broth (Oxoid) with 15% glycerol at -80˚C for further analysis.

**Fig. 1:**
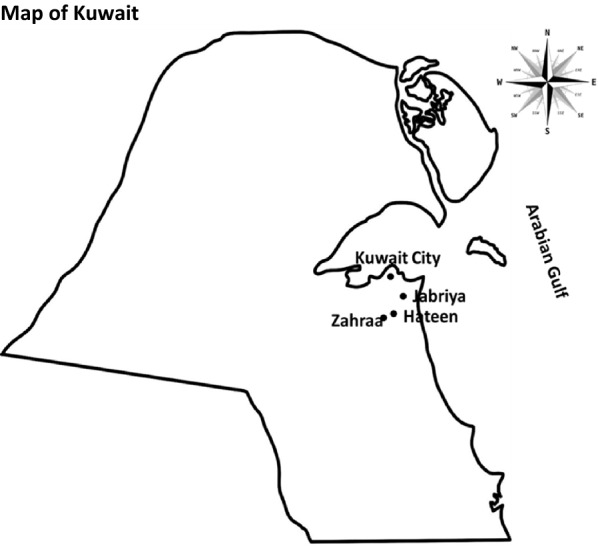
Locations of Jabriya, Zahraa and Hateen where sewage sampling was done. The map is not drawn to scale

There were some procedures that were common to all PCR assays. These were preparation of template DNA, agarose gel electrophoresis, and detection of amplicons. These are described below. Boiling method was used for DNA extraction. A loopful of *E. coli* colonies from MacConkey agar was boiled in PCR-grade water (ThermoFisherScientific) in an Eppendorf tube (Eppendorf) for 10 min. After cooling the tube on ice for 5 min, it was centrifuged at 12,000 rpm for 10 min in a Spectrafuge 24D centrifuge (Labnet). The DNA present in the supernatant was used as the template in the PCR assay. The concentration of DNA was measured using a NanoDrop spectrometer (ThermoFisherScientific). The amplification blend was made by mixing the following reagents in a PCR tube (Eppendorf) in a 10 µl reaction volume: 2 µl of master mix (Solis BioDyne), 1 µl of forward primer (10 pmol), 1 µl of reverse primer (10 pmol), 5 µl of PCR-grade H_2_O, and 1 µl of diluted DNA (25 ng). This PCR mixture was then placed in a thermal cycler (Applied Biosystems) for DNA amplification with the desired cycles. The primer sequences, cycling conditions, and amplicon sizes for various PCR assays are given in Additional file 1: Table S1 at https://github.com/MahdiRedha/GP-AdditionalFile-1Revised14Feb2022/blob/main/GP-AdditionalFile%201Revised14Feb2022.pdf. After amplification, the amplified products were separated by agarose gel electrophoresis. The concentration of the agarose in the gel was 1.0% (for products ˃800 bp), 1.5% (for products 200–800 bp) and 2% (for products < 200 bp). Electrophoresis of the products in the gel incorporated with ethidium bromide was carried out in Tris–Borate-EDTA buffer at a voltage of 100 V for ⁓ 40 min, and the gel was photographed under the UV light to detect amplicons.

Bacterial isolates identified as *E. coli* by API-20E test were subjected to a confirmatory PCR assay to detect universal stress protein gene (*uspA*) that is unique for *E. coli* [5]. Phylogenetic grouping of *E. coli* was performed by both the triplex and the quadruplex PCR methods [6]. The five categories of DEC were detected by PCR assays for the genes specified in Additional file 1: Table S1. ExPEC was identified by detecting the following genes by PCR assays: *vat* (vacuolating auto-transporter toxin), *fyuA* (ferric yersiniabactin uptake A)*, chuA* (coli heme utilization gene A)*,* and *yfcV* (yersinia fimbrial chaperon V) (Additional file 1: Table S1) by the criteria of Spurbeck et al. [7]; isolates positive for all four genes (*vat*, *fyuA*, *chuA*, *yfcV*) or the following three gene combinations: *fyuA* + *chuA* + *yfcV*, *fyuA* + *vat* + *chuA*, and *fyuA* + *yfcV* + *vat* were considered as ExPEC.

Of a total of 144 isolates identified as *E. coli* by API-20E test, 140 isolates were positive by *uspA* PCR and hence confirmed as *E. coli.* The number of *E. coli* cultured each month varied from 3 to 24. *E. coli* was cultured from Jabriya and Zahraa for all 12 months, and from Hateen for 11 months, except in May 2018 (Additional file 1: Table S2). The proportions of *E. coli* cultured from the three sites sampled were similar, being 32.8% (46 isolates) in Jabriya, 28.6% (40 isolates) in Zahraa, and 38.6% (54 isolates) in Hateen.

The results of phylogenetic analyses are shown in Additional file 1: Table S2. By triplex PCR grouping, the 140 isolates were classified into group A (68 isolates [48.6%]), group B1 (34 isolates [24. 3%]), group B2 (8 isolates [5.7%]), and group D (27 isolates [19.3%]). As required, isolates from group A and group D were further subjected to quadruplex PCR grouping. By quadruplex PCR, 68 group A isolates were reclassified into group A (65 isolates) and group C (3 isolates); 27 group D isolates were reclassified into group D (15 isolates), group E (4 isolates), and group F (8 isolates). After quadruplex PCR grouping, the rank order of the groups was: A (65 isolates [46.4%]) > B1(34 isolates [24.3%]) > D (15 isolates [10.7%]) > B2 and F (8 isolates [5.7%] each) > E (4 isolates [2.9%]) > C (3 isolates [2.1%]).

The different categories of *E. coli* were: ExPEC (14 isolates [10%]), DEC (3 isolates [2.1%]) and nonpathogenic *E. coli* (123 isolates [87.9%]). Further characterization is as below. Of the three DEC, two were atypical EPEC (*eae*^+^*bfp*^−^) (one cultured from June 2018 sample from Jabriya [isolate no. J28], and the other cultured from November 2018 sample from Hateen [isolate no. H164]), and one was an *ltA* + ETEC (cultured from July 2018 sample from Hateen [isolate no. H51]). The distribution of the genes that defines 14 isolates as ExPEC is shown in Table 1. The isolates had all four genes and two different combinations of three genes. The distribution of ExPEC according to date and site of collection is shown in Additional file 1: Table S3. ExPEC was isolated from all three sites except for the months of May, June, September, November, and December 2018 when it was not isolated from any site. The rank order of distribution of phylotypes in ExPEC was B2 (8 isolates [57.1%] > F (5 isolates [35.7%] > B1 (1 isolate [7.1%]). Nonpathogenic *E. coli* (not belonging to DEC or ExPEC), was in group A (65 isolates [52.9%]), B1 (32 isolates [26.0%]), D (14 isolates [11.4%]), E (4 isolates [3.3%]), C and F (3 isolates each [2.4%]), and B2 (2 isolates [1.6%]).

**Table 1:** Distribution of genes in *E. coli* isolates which define ExPEC, and phylotypes of ExPEC

ExPEC gene combination	No. of isolates positive	Phylotypes (no. of isolates)
*fyuA* + *yfcV* + *chuA*	6	B2 (1), F (5)
*vat* + *fyuA* + *yfcV* + *chuA*	5	B2 (5)
*vat* + *yfcV* + *chuA*	3	B1 (1), B2 (2)

*E. coli* bacteria are heterogeneous and constitute commensals, intestinal pathogens, and extra-intestinal pathogens. Grouping of *E. coli* can be done using molecular methods. One such method is the PCR phylogenetic typing done in our study. This typing method was developed by Clermont et al. [6]. They initially did the typing with a triplex PCR method using three genes (*chuA*, *yjaA*, and TspE4.C2), and assigned *E. coli* to four groups: A, B1, B2, and D [6]. When they compared the typing results with those of the multi-locus sequence typing, it was found that assigning some isolates to groups A and D was not accurate. Therefore, they developed a modified method, a quadruplex PCR method [6]. Quadruplex method uses modified primers targeting the same three genes (*chuA*, *yjaA*, and TspE4.C2), thus eliminating some problems associated with mismatches of the primers [6]. It also uses primers to target an additional gene, *arpA*. By this quadruplex PCR, some isolates of phylogroups A and D were further divided into groups C, E and F in our study. It was reported that quadruplex PCR also identifies cryptic clades I to V, which were not found in our study. Cryptic *E. coli* clades are extremely rare in human samples. They are mainly found in feces of animals, non-human mammals, and birds [8]. The proportion of reclassified *E. coli* phylogroups A and D in our study, was as predicted by Clermont et al*.* [6]. It has been shown that the PCR phylogenetic grouping has similar discriminatory power as enterobacterial repetitive intergenic consensus (ERIC) PCR and the laborious pulsed-field gel electrophoresis (PFGE) [9].

Only three of the 140 *E. coli* isolates belonged to DEC (2.1%). This low prevalence of DEC is in line with a study in children in Kuwait [10]. ExPEC pathotype is the causative agent of urinary tract infection, bacteremia/septicemia, and meningitis. It harbors the highest number of virulence factors that include adhesins, toxins, siderophores and hemolysins [11]. In one study [9], after final treatment of the sewage, the effluent had *E. coli* that harbored virulence factors specific for ExPEC suggesting that ExPEC strains can survive sewage treatments.

ExPEC strains do not have a defined set of genes as in the case of DEC. However, studies have shown that there are some genes that are commonly found in ExPEC pathotype in high frequencies, differentiating ExPEC from DEC and commensals. Spurbeck et al*.* [7] showed that four genes—*vat*, *fyuA, chuA,* and *yfcV*—could be used to identify uropathogenic *E. coli* (UPEC) isolates. By their criteria, isolates positive for all four genes (*vat*, *fyuA*, *chuA*, *yfcV*) or the following combinations of genes—*fyuA* + *chuA* + *yfcV*, *fyuA* + *vat* + *chuA*, and *fyuA* + *yfcV* + *vat*—were ExPEC. Isolates with these genes were found to have more virulence determinants and could colonize the bladder more effectively. The study of Johnson et al*.* [12] found that the following genes—*papA/papC*, *sfa*/*focDE*, *afa*/*draBC*, *iutA*, and *kpsMT* II—were highly associated with ExPEC isolates. Screening a collection of *E. coli* isolates by the tests of Spurbeck et al*.* [7] and Johnson et al*.* [12] showed that the isolates predicted as UPEC or ExPEC had most additional virulence genes [7]. Another study done by Johnson et al. [13] suggested that the tests of Spurbeck et al*.* [7] were valid for fecal isolates. Thus, it appeared that we could either use the criteria of Spurbeck et al. [7] or those of Johnson et al. [12] to define ExPEC. We chose the criteria of Spurbeck et al. [7] in our study. The four genes used in our study to define ExPEC appeared to correlate with virulence in a mouse model [7]. Thus, our criteria for detecting ExPEC have a high reliability compared to criteria used in other studies. To our knowledge, ours is the first study, where these four genes were used to identify ExPEC from sewage.

In previous studies, most of the ExPEC belonged to groups B2 and D, while those *E. coli* that belonged to group A or B1 were recognized as commensals [14, 15]. It was also demonstrated that isolates from group B2 were more virulent than the isolates from the other groups [15]. In our study too, more than half of the ExPEC isolates belonged to group B2.

Some studies have suggested that strains belonging to groups A and B1 may also harbor virulence traits that enable them to survive and proliferate in the harsh environments outside the gastrointestinal tract [16]. Some of these strains may cause intestinal and extra-intestinal illnesses as they possess the required virulence genes. Thus, the two groups, A and B1, should not be dismissed as non-pathogenic [10, 17].

Probably, a limitation of our study is its complete reliance on PCR methods for detection of virulence genes. The four genes that were used for the detection of ExPEC were based on the expression of these genes in in vivo models by the original investigators [7]. We are not aware of any studies that showed a lack of expression or non-functionality of these genes due to mutation. Even so, a future study should look at the expression of these virulence genes that define ExPEC, in isolates from sewage. Therefore, the observations of our study may be considered as preliminary.

The characteristics of *E. coli* in raw sewage in Kuwait reflects *E. coli* population in the colonic flora of the Kuwaiti population. The finding of a relatively high prevalence of ExPEC in raw sewage in Kuwait is of a public health concern for the use of coastal waters for recreation. A future study that examines the carriage of ExPEC virulence genes by *E. coli* isolates recovered from contaminated coastal waters is warranted. The dilution of the organisms by the sea water as a mitigating factor must also be taken into consideration.

## Supplementary Information

**Additional file 1**: ** Table S1**. List of genes, sequences of primers, cycling conditions for PCR assays, and amplicon sizes.** Table S2**. Sample collection dates and locations, and phylogenetic groupings of E. coli by triplex and quadruplex PCR assays.** Table S3**. Distribution of ExPEC according to location and date of collection of samples.

## Data Availability

All data generated or analyzed during this study are included in this published article [and its supplementary information file].

## Abbreviations

DEC: Diarrheagenic *E. coli*
EAEC: Enteroaggregative *E. coli*
STEC: Shiga toxin-producing *E. coli*
EIEC: Enteroinvasive *E. coli*
EPEC: Enteropathogenic *E. coli*
ETEC: Enterotoxigenic *E. coli*
ExPEC: Extra-intestinal pathogenic *E. coli*
UPEC: Uropathogenic *E. coli*
