# Supplementary material for: Virulence and phylogenetic groups of Escherichia coli cultured from raw sewage in Kuwait
Source: Gut Pathog. 2022 Apr 26;14:18. doi: 10.1186/s13099-022-00490-4 (PMC9044736; doi:10.1186/s13099-022-00490-4)
Supplement: Supplementary file 1 — Additional file 1: Table S1. List of genes, sequences of primers, cycling conditions for PCR assays, and amplicon sizes. Table S2. Sample collection dates and locations, and phylogenetic groupings of E. coli by triplex and quadruplex PCR assays. Table S3. Distribution of ExPEC according to location and date of collection of samples. [file 13099_2022_490_MOESM1_ESM.pdf]

Additional File 1

Table S1. List of genes, sequences of primers, cycling conditions for PCR assays, and amplicon sizes.

Assay	Gene	Primer sequence	PCR program[a]	Product size (bp)	Reference
E. coli confirmation	*uspA*	F: 5’-CCGATACGCTGCCAATCAGT-3’	94°C/ 120 s, 70°C/ 60 s, 72°C/ 60 s (30 cycles)	884	1
		R: 5’-ACGCAGACCGTAGGCCAGAT-3’			
Phylogenetic typing (Triplex method)	*chuA*	F: 5’-GACGAACCAACGGTCAGGAT-3’	94°C/ 30 s, 55°C/ 30 s, 72°C/ 30 s (30 cycles)	279	2
		R: 5’-TGCCGCCAGTACCAAAGACA-3’			
	yjaA	F: 5’-TGAAGTGTCAGGAGACGCTG-3’		211	
		R: 5’-ATGGAGAATGCGTTCCTCAAC-3’			
	TspE4.C2	F: 5’-GAGTAATGTCGGGGCATTCA-3’		152	
		R: 5’-CGCGCCAACAAAGTATTACG-3’			
Phylogenetic typing (Quadruplex method)	*chuA*	F: 5’-ATGGTACCGGACGAACCAAC-3’	94°C/ 5 s, 57°C/ 20 s (30 cycles)	288	3
		R: 5’-TGCCGCCAGTACCAAAGACA-3’			
	yjaA	F: 5’-CAAACGTGAAGTGTCAGGAG-3’		211	
		R: 5’-AATGCGTTCCTCAACCTGTG-3’			
	TspE4.C2	F: 5’-CACTATTCGTAAGGTCATCC-3’		152	

		R: 5′-AGTTTATCGCTGCGGGTCGC-3′			
	arpA (Group F)	F: 5′-AACGCTATTCGCCAGCTTGC-3′		400	
		R: 5′-TCTCCCCATACCGTACGCTA-3′			
	arpA (Group E)	F: 5′-GATTCCATCTTGTCAAAATATGCC-3′		301	
		R: 5′-GAAAAGAAAAAGAATTCCCAAGAG-3′			
	trpA (Group C)	F: 5′-AGTTTTATGCCCAGTGCGAG-3′	94°C/ 5 s, 59°C/ 20 s (30 cycles)	219	
		R: 5′-TCTGCGCCGGTCACGCCC-3′			
	trpA (Internal control)	F: 5′-CGGCGATAAAGACATCTTCAC-3′	94°C/ 5 s, 57°C/ 20 s (30 cycles)	489	
		R: 5′-GCAACGCGGCCTGGCGGAAG-3′			
DEC[b]	*eae*	F: 5′-GACCCGGCACAAGCATAAGC-3′	95°C/ 30 s, 54°C/ 90 s, 72°C/ 90 s (30 cycles)	384	4, 5
		R: 5′-CCACCTGCAGCAACAAGAGG-3′			
	stx1	F: 5′-ATAAATCGCCATTCGTTGACTAC-3′	95°C/ 30 s, 52°C/ 60 s, 72°C/ 60 s (35 cycles)	180	
		R: 5′-AGAACGCCCACTGAGATCATC-3′			
	stx2	F: 5′-GGCACTGTCTGAAACTGCTCC-3′		255	
		R: 5′-TCGCCAGTTATCTGACATTCTG-3′			

	ltA	F: 5’-GGCGACAGATTATACCGTGC-3’	94°C/ 60 s, 50°C/ 60 s, 72°C/ 120 s (35 cycles)	696	
		R: 5’-CCGAATTCTGTTATATATGTC-3’			
	st1A	F: 5’-TCTGTATTATCTTTCCCCTC-3’		186	
		R: 5’-ATAACATCCAGCACAGGC-3’			
	ipaH	F: 5’-GTTCCTTGACCGCCTTTCCGATACCGTC-3’	94°C/ 40 s, 60°C/ 60 s, 72°C/ 60 s (35 cycles)	620	
		R: 5’-GCCGGTCAGCCACCCTCTGAGAGTAC-3’			
	bfpA	F: 5’-ATTGAATCTGCAATGGTGC-3’	95°C/ 40 s, 55°C/ 40 s, 72°C/ 40 s (30 cycles)	461	
		R: 5’-ATAGCAGTCGATTTAGCAGCC-3’			
	aggA	F: 5’-ATGCATTACTTTGGGTTTAG-3’	94°C/ 60 s, 50°C/ 60 s, 72°C/ 120 s (35 cycles)	414	
		R: 5’-TCAACCTTGACACTTGCC-3’			
ExPEC[c]	*vat*	F: 5’-TCAGGACACGTTCAGGCATTCAGT-3’	94°C/ 30 s, 63°C/ 90 s, 72°C/ 90 s	1100	6
		R: 5’-GGCCAGAACATTTGCTCCCTTGTT-3’			
	fyuA	F: 5’-GTAAACAATCTTCCCGCTCGGCAT-3’		850	

		R: 5’-TGACGATTAACGAACCGGAAGGGA-3’	(30 cycles)		
	chuA	F: 5’-CTGAAACCATGACCGTTACG-3’		652	
		R: 5’-TTGTAGTAACGCACTAAACC-3’			
	yfcV	F: 5’-ACATGGAGACCACGTTCACC-3’		292	
		R: 5’-GTAATCTGGAATGTGGTCAGG-3’			

[a]Before starting the PCR cycle, DNA was first denatured at 95°C/15 min. After completion of the cycle, there was a final primer extension at 72°C/8 min.

[b]Diarrheagenic *E. coli* are defined by the following genes: *eae*+*bfpA*/*eae* (EPEC), *ltA*+*st1A*/*ltA*/*st1A* (ETEC), *ipaH* (EIEC), *eae*+*stx1*+*stx2*/*stx1*/*stx2* (STEC), and *aggA* (EAEC).

[c]Extra-intestinal pathogenic *E. coli*

References in Table 1

1. Chen J, Griffiths MW. PCR differentiation of *Escherichia coli* from other Gram-negative bacteria using primers derived from the nucleotide sequences flanking the gene encoding the universal stress protein. Lett Appl Microbiol. 1998; 27: 369–71.

2. Clermont O, Bonacorsi S, Bingen E. Rapid and simple determination of the *Escherichia coli* phylogenetic group. Appl Environ Microbiol. 2000; 66: 4555–8.

3. Clermont O, Christenson JK, Denamur E, Gordon DM. The Clermont *Escherichia coli* phylo-typing method revisited: Improvement of specificity and detection of new phylo-groups. Environ Microbiol Rep. 2013; 5: 58–65.

4. Robins-Browne RM, Bordun A-M, Tauschek M, Bennett-Wood VR, Rusell J, Oppedisano F, et al. *Escherichia coli* and community-acquired gastroenteritis, Melbourne, Australia. Emerg Infect Dis. 2004; 10: 1797-1805.

5. Sethabutr O, Venkatesan M, Yam S, Pang LW, Smoak BL, Sang WK, et al. Detection of PCR product of the *ipaH* gene from *Shigella* and enteroinvasive *Escherichia coli* by enzyme-linked immunosorbent assay. Diagn Microbiol Infect Dis. 2000; 37: 11-16.

6. Spurbeck RR, Dinh PC Jr, Walk ST, Stapleton AE, Hooton TM, Nolan LK, et al. *Escherichia coli* isolates that carry *vat*, *fyuA*, *chuA*, and *yfcV* efficiently colonize the urinary tract. Infect Immun. 2012; 80: 4115–22.

Table S2. Sample collection dates and locations, and phylogenetic groupings of *E. coli* by triplex and quadruplex PCR assays.

Date of sample collection	Location (no. of confirmed *E. coli*)	Phylogenetic grouping by triplex PCR (no. of isolates)	Phylogenetic grouping by quadruplex PCR (no. of isolates)
14 May 2018	J[a] (1)<br>Z[b] (2)	B1 (2)<br>D (1)	B1 (2)<br>D (1)
11 June 2018	J (7)<br>Z (2)<br>H[c] (5)	A (6)<br>B1 (3)<br>D (5)	A (6)<br>B1 (3)<br>D (4)<br>E (1)
9 July 2018	J (1)<br>Z (3)<br>H (2)	A (3)<br>B1 (1)<br>B2 (1)<br>D (1)	A (3)<br>B1 (1)<br>B2 (1)<br>D (1)
13 August 2018	J (6)<br>Z (6)<br>H (7)	A (9)<br>B1 (6)<br>B2 (2)<br>D (2)	A (9)<br>B1 (6)<br>B2 (2)<br>D (1)<br>E (1)
10 September 2018	J (2)<br>Z (1)<br>H (9)	A (8)<br>B1 (3)<br>B2 (1)	A (7)<br>B1 (3)<br>B2 (1)<br>C (1)

8 October 2018	J (4)<br>Z (1)<br>H (4)	A (4)<br>B1 (3)<br>D (2)	A (4)<br>B1 (3)<br>D (1)<br>F (1)
12 November 2018	J (4)<br>Z (1)<br>H (4)	A (7)<br>B1 (2)	A (7)<br>B1 (2)
10 December 2018	J (3)<br>Z (4)<br>H (3)	A (3)<br>B1 (3)<br>D (4)	A (3)<br>B1 (3)<br>D (2)<br>F (2)
7 January 2019	J (4)<br>Z (5)<br>H (5)	A (3)<br>B1 (3)<br>B2 (2)<br>D (6)	A (3)<br>B1 (3)<br>B2 (2)<br>D (3)<br>F (1)<br>E (2)
4 February 2019	J (4)<br>Z (2)<br>H (2)	A (3)<br>B1 (3)<br>D (2)	A (3)<br>B1 (3)<br>F (2)
11 March 2019	J (3)<br>Z (6)<br>H (3)	A (6)<br>B1 (1)<br>B2 (4)<br>D (1)	A (6)<br>B1 (1)<br>B2 (4)<br>D (1)
8 April 2019	J (7)<br>Z (7)<br>H (10)	A (16)<br>B1 (5)<br>D (3)	A (14)<br>B1 (5)<br>D (1)<br>F (2)<br>C (2)

[a]Jabriya.

[b]Zahraa.

[c]Hateen.

Table S3. Distribution of ExPEC according to location and date of collection of samples.

Month/Year	No. of ExPEC isolates	Location		
		Jabriya	Zahraa	Hateen
14 May/2018	0	0	0	0
11 June/2018	0	0	0	0
9 July/2018	1	0	1	0
13 August/2018	2	0	0	2
10 September/2018	0	0	0	0
8 October/2018	1	1	0	0
12 November/2018	0	0	0	0
10 December/2018	0	0	0	0
7 January/2019	2	1	1	0
4 February/2019	3	0	1	2
11 March/2019	4	2	1	1
8 April/2019	1	1	0	0
